# Gender Differences Regarding the Impact of Math Anxiety on Arithmetic Performance in Second and Fourth Graders

**DOI:** 10.3389/fpsyg.2018.02690

**Published:** 2019-01-18

**Authors:** Hanneke I. Van Mier, Tamara M. J. Schleepen, Fabian C. G. Van den Berg

**Affiliations:** Department of Cognitive Neuroscience, Developmental Cognitive Neuroscience Section, Faculty of Psychology and Neuroscience, Maastricht University, Maastricht, Netherlands

**Keywords:** math anxiety, gender, mathematics, elementary school, children, arithmetic

## Abstract

The development of math skills is crucial for adequate functioning in academic and professional settings as well as in daily life. A factor that has been shown to negatively influence performance and acquisition of math skills is math anxiety. With the high prevalence of math anxiety in society and the long lasting effects on math performance, it is important to study the relation between math anxiety and math performance in young children. Since math anxiety is often more pronounced in women than in men, it is essential to take the effect of gender into account. While the effect of gender on the relation between math anxiety and math performance has been studied in adults and adolescents, less research has focused on children, especially children at young ages. To fill this gap, the current study examined how the relation between math anxiety and math performance differed between boys and girls in early elementary school years. Math anxiety and math performance was assessed in 124 second- and fourth-grade children (67 girls and 57 boys). Although boys and girls showed more or less equal levels of math anxiety and performed similarly at the arithmetic task, correlation analyses showed that only in girls, math anxiety significantly correlated with math performance. Analyses investigating if math anxiety moderated the effect of gender and grade on math performance revealed significant differences between boys and girls. Higher levels of math anxiety only significantly and negatively moderated math performance in girls, with the greatest effect observed in 2nd grade girls. These findings highlight the importance of taking gender differences into account when studying the effect of math anxiety. The results showed that math anxiety is already negatively linked to math performance in girls as early as second grade. The present findings emphasize the importance of the early identification and remediation of math anxiety in girls to prevent long lasting effects. Possible causes for the gender related differences will be discussed.

## Introduction

Developing and having adequate numerical and mathematical skills is a prerequisite to function effectively in daily life and influences participation and success in many careers especially those in the fields of science, technology, engineering, and mathematics (STEM) (Beilock and Maloney, 2015; Ferguson et al., 2015). Ashcraft and Kirk (2001) reported that about 20% of the population shows a more or less severe negative affective reaction in situations that involve numerical and mathematical activities which is referred to as math anxiety (MA). It is not said that high math anxiety is always related to poor math performance or the other way around. Devine et al. (2018) found that in a sample of almost 1,800 elementary and secondary school children, 77% of the children with high math anxiety had normal to high math scores. Only around 20% of the children with poor math scores had high math anxiety. However, it can be devastating if math performance is hindered by affective rather than cognitive factors (Ashcraft and Moore, 2009; Else-Quest et al., 2010), resulting in a failure to perform at one's best level. It is therefore important to recognize math anxiety in younger children to prevent that they develop an avoidance of mathematics which might have lifelong effects (Aarnos and Perkkilä, 2012). This might be in particular important for girls considering the fact that women are underrepresented in STEM careers (Else-Quest et al., 2010; Wang and Degol, 2017). To evaluate if math anxiety differentially impacted math performance in boys and girls we studied this effect in children in early elementary school years in second and fourth grade.

It has consistently been reported that higher levels of math anxiety are related to lower scores on math achievement tests, fewer math courses taken, lower grades in math courses, and to avoiding certain career paths involving mathematics (e.g., Ma, 1999; Ashcraft and Krause, 2007; Ashcraft and Moore, 2009). Math anxiety can be provoked by simple arithmetic problems when performing under time pressure (Ashcraft, 2002; Caviola et al., 2017a) and with increasing complexity of arithmetic tasks (Kellogg et al., 1999; Ashcraft and Krause, 2007). Although the relation between math anxiety and achievement in mathematics has been studied extensively in adults and adolescents, little research has focused on this relation in young children in elementary school (Ganley and McGraw, 2016). Most studies involving adults and adolescents reported significant negative correlations between math anxiety scores and math performance (e.g., Hembree, 1990; Ma, 1999; Ashcraft and Kirk, 2001; Hill et al., 2016) as did studies involving elementary school children in fourth grade or beyond (Suinn et al., 1988; Chiu and Henry, 1990; Erturan and Jansen, 2015; Schleepen and Van Mier, 2016; Devine et al., 2018). Although studies involving children in early primary school grades are limited, they showed that math anxiety can already be observed in children as young as 6 – 7 years regardless of ethnical background (Aarnos and Perkkilä, 2012; Harari et al., 2013; Ramirez et al., 2013; Kucian et al., 2018). Research has also shown that math anxiety correlated negatively with math performance already in grade 2 and that this relation was independent of other anxiety measures (Wu et al., 2012; Cargnelutti et al., 2017). An fMRI study in which 7- to 9-year-old children had to verify addition and subtraction problems, showed that math anxiety was associated with increased activity in regions that play a role in the processing of negative emotions, while activity in regions involved in mathematical reasoning was decreased (Young et al., 2012). Most studies involving children in early elementary school years showed a negative relation between math anxiety and math performance (Aarnos and Perkkilä, 2012; Wu et al., 2012; Cargnelutti et al., 2017 (in 3^rd^ graders); Caviola et al., 2017b; Kucian et al., 2018) or on the use of math problem solving strategies (Ramirez et al., 2016). Contrasting findings have been reported by others [(Krinzinger et al., 2009; Cargnelutti et al., 2017) (for 2nd graders)] who did not find a relation between math anxiety and math performance in younger children.

The majority of studies addressing gender differences in adults regarding levels of math anxiety have shown that women reported higher levels than men (Hembree, 1990; Miller and Bichsel, 2004; Ferguson et al., 2015; Jansen et al., 2016). Based on reports by Hembree (1990) and Hopko (2003), Ashcraft and Moore (2009) calculated that females score approximately 0.3 standard deviation (SD) higher on math anxiety scales than men from grade 6 through college, with math anxiety levels peaking at grades 9 to 10. Significant gender differences regarding math anxiety were found in junior and senior high school students, with girls reporting higher levels of math anxiety than boys (Else-Quest et al., 2010; Goetz et al., 2013; Hill et al., 2016). In a longitudinal study math anxiety levels and math performance were studied in boys and girls from grade 7 to grade 12 (Ma and Xu, 2004). The authors found that stability effects for math anxiety were significantly stronger for girls than for boys, suggesting that math anxiety is passed on from year to year from junior to senior high school among girls. This implies that when girls develop math anxiety during early (high) school years, there is a tendency that the anxiety is sustained regardless of math performance. The majority of studies involving elementary school children report an absence of gender differences regarding math anxiety scores (Chiu and Henry, 1990; Young et al., 2012; Harari et al., 2013; Ramirez et al., 2013; Erturan and Jansen, 2015; Schleepen and Van Mier, 2016; Kucian et al., 2018).

Although scarce, studies addressing gender differences in the relation between math anxiety and math scores in late elementary and early secondary school children showed that this relation was only significant in girls (Devine et al., 2012; Erturan and Jansen, 2015; Schleepen and Van Mier, 2016). Hill et al. (2016) found a significant correlation in girls but not in boys (grade 3–5, mean ages 9:5 years). So far no studies have investigated possible gender differences in the relation between math anxiety and arithmetic performance in early elementary school children. To address this gap in the literature, the current study examined if math anxiety differentially affects math performance in girls and boys. We addressed this question in a larger and younger sample than in the study of Schleepen and Van Mier (2016) by including children from second and fourth grade. In the current study mathematical performance was measured by the Tempo Test Arithmetic (TTA). This test measures the production of basic arithmetic performance (De Vos, 1992). Children have to perform under time pressure by solving as many problems possible within a certain time period. We used this test because grades for math in schools are almost always based on performance in tests that have to be finished within a certain time period. Time pressure can work as a stressor and as such might elicit an online anxiety state during task execution resulting in less optimal strategy selection and lower math performance (Caviola et al., 2017a). Several studies have included the TTA to measure arithmetic ability in children (e.g., Van der Sluis et al., 2005; Stock et al., 2009) also in relation to math anxiety (Erturan and Jansen, 2015; Schleepen and Van Mier, 2016).

It has been found that math anxiety correlates moderately with general anxiety (Dreger and Aiken Jr, 1957; Hill et al., 2016) and with test anxiety (Dew et al., 1984; Hembree, 1990). Because these correlations were much lower than the correlations between different math anxiety measures, Hembree (1990) therefore suggested that math anxiety is a unique and different construct. However, to rule out any effect of test anxiety on math anxiety it has been advised to control for the former (Devine et al., 2012; Eden et al., 2013). When the effect of test anxiety was controlled for in partial correlation analyses, Devine et al. (2012) still found a highly significant correlation between math anxiety and math performance in girls, while it was only marginally significant in boys. For that reason we included a measure of test anxiety in our study, although we have to state that we only had the option to measure test anxiety in the youngest group of participants, the children in grade two. Because the children of grade four performed additional tests, which are beyond the scope of this paper, there was no room/time to add a measure of test anxiety in this group.

To summarize, math anxiety seems to be higher in females than in males, although gender related differences regarding math performance are small or non-existent. Usually a significant negative link between math anxiety and math performance is reported. Studies addressing gender differences regarding this link showed that it was only significant for girls and women. These findings are mainly based on studies involving children in late elementary or secondary school and adults. The current study aimed at establishing if gender differences in the relation between math anxiety and math performance are already present in children in early elementary school years. Correlation analyses per gender and grade were performed to study this relation. A linear mixed model was performed to study the moderation effect of math anxiety on math performance for gender, grade and the interaction of gender by grade. Based on the few studies involving younger children, we hypothesized that math anxiety would negatively correlate with and moderate math performance mainly in girls.

## Methods

### Participants

Participants were 124 elementary school children, of which 62 children attended grade two (mean age 8.13, *SD* = 0.61) and 62 children grade four (mean age = 9.55, *SD* = 0.65). Gender was balanced in the grade four group with 31 girls (mean age = 9.61, *SD* = 0.67) and 31 boys (mean age = 9.48, *SD* = 0.63). Regarding the second-graders, there was a slight gender imbalance in this group with 36 girls (mean age = 8.11, *SD* = 0.62) and 26 boys (mean age = 8.15, *SD* = 0.61), but this difference was not significant [*X*^2^ (1, *N* = 62) = 1.61, *p* = 0.20]. For all children included in the study written informed parental consent was obtained before the start of the study. None of the children had a history of learning difficulties or was diagnosed with any developmental disorder. Children were recruited from different elementary schools, which were all located in suburban areas in the southern part of the Netherlands. The majority of the children were Caucasian and from middle class families. Approval for the study was obtained by the ethical committee of the Faculty of Psychology and Neuroscience of Maastricht University. The study was performed in accordance with the ethical standards as laid down in the 1964 Declaration of Helsinki.

### Materials

#### Math Performance

Math performance was measured by means of the Tempo Test Arithmetic (TTA; Tempo Toets Rekenen; De Vos, 1992). The TTA is a speeded test measuring arithmetic ability. It is a standardized paper and pencil achievement test, used at elementary schools to measure automaticity and progress on basic arithmetic problems. The test consists of five columns with each column containing 40 arithmetic problems of increasing difficulty, resulting in a total of 200 problems. Each column covers a different arithmetical operation: addition, subtraction, multiplication, and division. The last column consists of a mixture of the four earlier mentioned operations. Although originally presented in five rows on one sheet of paper, in the current study each column was printed on a separate sheet of paper, and had to be filled out from top to bottom. Children were instructed to solve as many problems as possible within a time period of 1 min per column. The number of correctly answered problems in a column is the total score for that column. Higher scores are representative of higher levels of math competence.

#### Math Anxiety

Math anxiety was measured with a Dutch translation of the revised Child Math Anxiety Questionnaire (CMAQ-R; Maloney et al., 2015; Ramirez et al., 2016), which is a revised version of the Child Math Anxiety Questionnaire (Ramirez et al., 2013), being suitable for children from first grade. This paper-and-pencil questionnaire consists of 16 items, describing a situation that relates to math. The situations are formulated in such a way that they are comprehensible for younger school aged children. Children were asked to answer how they feel when confronted with a certain math situation, for example: ‘How do you feel when you have to solve 27+15' or ‘How do you feel when you are being called on by a teacher to explain a math problem on the blackboard'. The children answered the questions by indicating their feeling on a slider with five faces, which contained a hidden 5-point Likert scale from 1 to 5 consisting of 1 (not nervous at all), 2 (a little nervous), 3 (somewhat nervous), 4 (very nervous), and 5 (extremely nervous). The scale featured a calm, smiley face on the far left, a semi nervous face in the middle, and a very, very nervous face on the far right. Scores ranged from 16 to 80, with higher scores indicating higher levels of math anxiety. The CMAQ-R showed good reliability, with Cronbach's alpha being 0.84 being measured in the fall semester and 0.82 being measured in the spring semester) (Maloney et al., 2015) and alpha being 0.83 (Ramirez et al., 2016). In the present sample using a Dutch translation, the internal consistency among the 16 items was 0.86 for the total sample, with an alpha of 0.85 for the 2nd graders and 0.86 for the 4th graders.

#### Test Anxiety

The level of test anxiety was measured with a Dutch translation of the Children's Test Anxiety Scale (CTAS; Wren and Benson, 2004) suited for children in grades 2 to 6. The CTAS is a self-report paper-and-pencil questionnaire containing 30 items that describe the feelings a child can experience during a test situation. The items repeatedly start with the sentence ‘While I am making a test……' followed by a statement. The items cover how children may think (13 items related to thought; e.g., “I worry about failing”), how they may feel (9 items related to autonomic reactions; e.g., “My hands shake”) and how they may act (8 items related to off-task behaviors; e.g., “I look around the room”). Children were asked to select the answer that best described how anxious they think, feel and act in a certain test situation or setting using a 1 to 4-point Likert scale with 1 indicating “almost never”, 2 indicating “some of the time,” 3 indicating “most of the time” and 4 indicating “almost always.” Adding up the scores of all items determined the test anxiety level. A higher score on the CTAS indicated a higher level of test anxiety, with a minimum score of 30 and a maximum score of 120. The CTAS showed good reliability coefficients and is considered a reliable instrument to measure test anxiety in children (Zeidner, 2007; Weissman et al., 2008; Nyroos et al., 2015; Korhonen et al., 2018), with Cronbach's alpha being 0.92 in the original and cross-validation sample of Wren and Benson (2004) and alpha being 0.89 in the studies of Nyroos et al. (2015) and Korhonen et al. (2018) using a Swedish translation of the CTAS. In the current sample of 2nd graders using a Dutch translation of the CTAS, the internal consistency was good with an alpha of 0.88.

### Procedure

All children were tested at school and testing was performed during regular school hours during the spring semester of the school year. All children received the same (verbal) instructions. The math anxiety (CMAQ-R) and test anxiety (CTAS) questionnaires were performed individually outside of the classroom in a quiet room. The CTAS was only performed by the children in grade 2. The children were sitting at a table with the researcher to the right of the child. The CMAQ-R and CTAS were filled out on paper. The researcher read the questions to the child and the child responded by pointing to the respective answer. The Tempo Test Arithmetic (TTA) was administered in the classroom with all children working on the tasks at the same time. Children were instructed to perform the TTA as quickly and accurately as possible. They did not receive any form of feedback on their performance.

Regarding the TTA, the children of grade 2 only performed columns 1 to 3 and the mixed column, involving only addition, subtraction and multiplication problems. Division problems were not tested in this group because children in this grade had just started with division problems. The mixed column (for this group referred to as column 4) also only included addition, subtraction and multiplication problems. Therefore in this group the maximum score that could have been obtained was 150 (40 problems for columns 1 to 3 and 30 problems for column 4). The children of grade 4 performed all columns and a maximum score of 200 could have been obtained.

Children performed additional tasks depending on the grade they were in, which were collected for different purposes and are not included in this paper. Because of time constraints due to more additional tasks, test anxiety was not measured in children in grade 4. The relevant tasks for this paper were in both grades administered in the same order. The CMAQ-R and CTAS (for children in grade 2) were administered before the TTA. Between tasks, children were given a short break. After completion of all tasks, the participating children were each rewarded with a small gift while the school/class received a monetary compensation.

## Results

### Descriptive Analyses

Descriptive statistics with means and standard deviations for the administered questionnaires and math test for boys and girls in grade 2 and 4 are displayed in Table 1 while averages per grade and gender can be seen in Table 2.

**Table 1:** Means and standard deviation (SD) for age, math, and test anxiety scores, and arithmetic test scores for boys and girls by grade.

	**Grade 2**		**Grade 4**	
	**Boys**	**Girls**	**Boys**	**Girls**
*N*	26	36	31	31
Age	8.15 (0.61)	8.11 (0.62)	9.48 (0.63)	9.61 (0.67)
CMAQ-R	32.8 (10.0)	35.8 (11.3)	27.0 (7.0)	30.3 (9.1)
CTAS	52.5 (12.5)	55.4 (14.0)	–	–
TTA				
Column 1 to 3	42.6 (8.8)	40.9 (11.5)	65.5 (15.8)	58.8 (13.4)
Column 1 to 4	52.7 (11.2)	50.7 (14.5)		
Column 1 to 5			101.0 (27.2)	89.6 (21.9)

*All scores represent raw scores*.

**Table 2:** Means and standard deviation (SD) for age, math anxiety scores, and arithmetic test scores for grade and gender.

	**Grade**		**Gender**	
	**Grade 2**	**Grade 4**	**Boys**	**Girls**
*N*	62	62	57	67
Age	8.13 (0.61)	9.55 (0.64)		
CMAQ	34.2 (10.7)	28.6 (8.2)	29.6 (8.9)	32.9 (10.5)
TTA score			
Column 1 to 3	41.6 (10.4)	62.2 (14.9)	55.1 (17.4)	49.2 (15.2)

*All scores represent raw scores*.

To study differences between genders and grades for the TTA scores, we additionally calculated the scores of the first 3 columns of the TTA because those columns were performed in the same way for all children in both grades and used those scores in the analysis. Because we did not have equal numbers of boys and girls in the 2nd grade group, we checked for homogeneity of variances. Levene's tests showed that homogeneity of variances was met for all variables; *F*_(1, 60)_ = 2.502, *p* = 0.12 for math anxiety, *F*_(1, 60)_ = 1.171, *p* = 0.28 for test anxiety, *F*_(1, 60)_ = 1.488, *p* = 0.23 for TTA columns 1 to 3, and *F*_(1, 60)_ = 1.083, *p* = 0.30 for TTA columns 1 to 4. We also checked for outliers in each group and found a total of four outliers, one outlier for math anxiety in the boys of grade 4, one for test anxiety in the boys of grade 2, and for the TTA we found one outlier for girls in grade 2 and one for girls in grade 4.

Two univariate ANOVA's with gender and grade as independent variables and math anxiety and TTA scores (columns 1 to 3) as dependent variables were performed to study the abovementioned differences. The analysis for math anxiety scores showed only a significant effect of grade [*F*_(1, 120)_ = 10.822, *p* < 0.01]. The effect of gender was not significant but showed a trend [*F*_(1, 120)_ = 3.318, *p* = 0.071]. The interaction of gender and grade was not significant [*F*_(1, 120)_ = 0.007, *p* = 0.933]. The same effects were found for the TTA scores with a significant effect of grade [*F*_(1, 120)_ = 79.401, *p* = 0.001], a trend effect of gender [*F*_(1, 120)_ = 3.388, *p* = 0.068] and a non-significant interaction of gender and grade [*F*_(1, 120)_ = 1.252, *p* = 0.265]. Excluding the outliers in the univariate ANOVA's resulted in a change from a trend effect to a significant effect of gender for the CMAQ-R [*F*_(1, 119)_ = 4.15, *p* < 0.05], with girls having significantly higher math anxiety scores than boys. The trend effect of gender for the TTA changed to not significant [*F*_(1, 119)_ = 2.185, *p* = 0.142]. The significances of the other effects or interactions did not change.

A significant effect of grade for the TTA scores was expected, because we used general scores for the TTA; children in grade 4 solved more problems, as was expected. Note the significantly higher math anxiety score of children in grade 2 (mean score of 34.2) compared to children in grade 4 (mean score of 28.6). The effect of gender showed a trend for both variables. As can be seen in Table 2, boys scored slightly higher on the TTA, but lower on the CMAQ-R than girls. Total score of the TTA including all columns performed per grade, showed that boys and girls in grade 2 did not perform significantly different on this test [*F*_(1, 60)_ = 0.336, *p* = 0.565] regarding the four columns, while there was a trend significant effect for gender in grade 4 concerning all five columns [*F*_(1, 60)_ = 3.273, *p* = 0.075]. Regarding test anxiety, there was no significant effect of gender on the score for children in grade 2 [*F*_(1, 60)_=0.668, *p*=0.417]. Excluding the boy with the outlier did not change the significance for the effect of gender.

**Figure 1:**
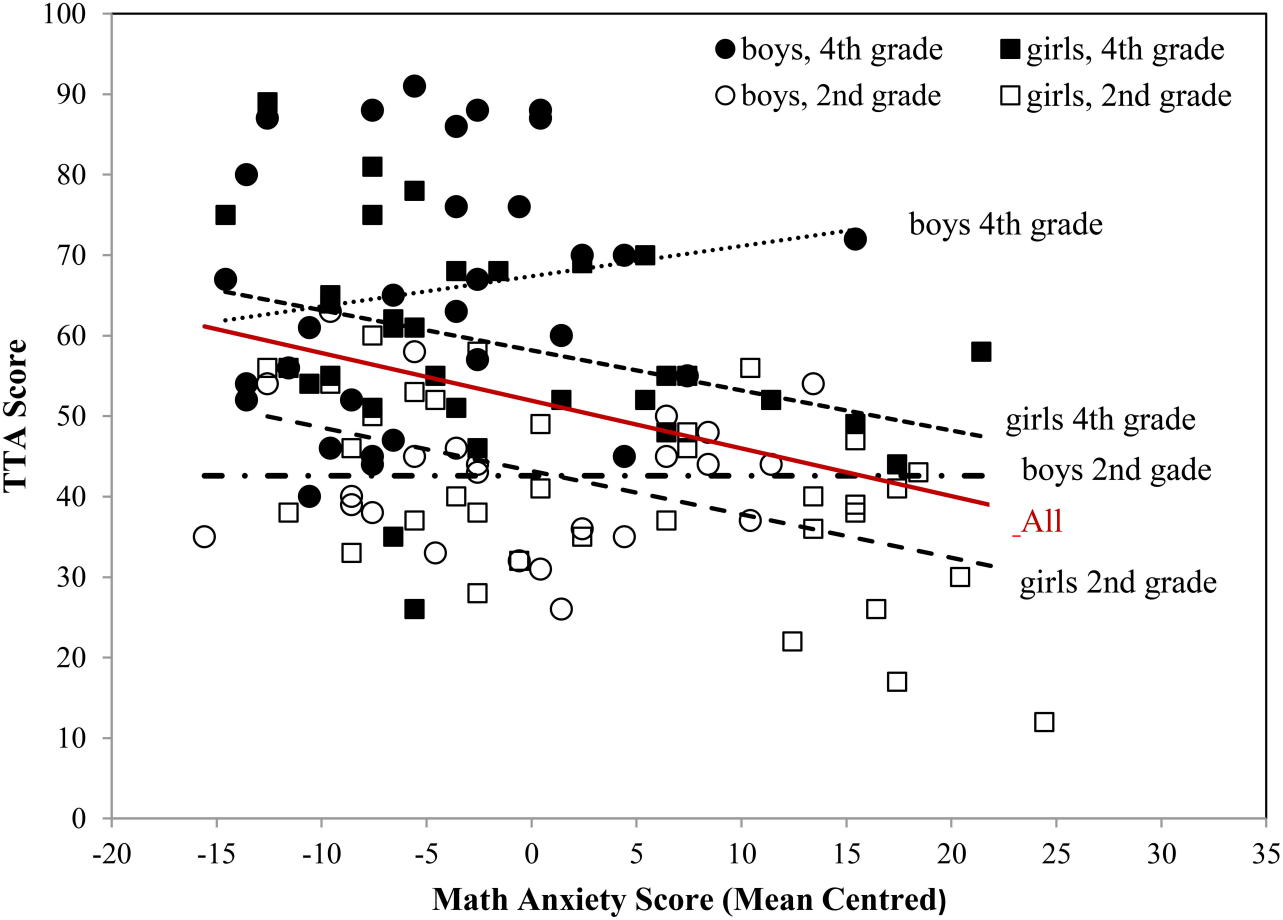
Math anxiety scores plotted against the TTA scores (columns 1 to 3) and slopes for grade and gender.

Figure 1 shows the math anxiety scores (mean centered) plotted against the TTA scores (columns 1 to 3) for grade and gender. A negative linear overall relationship between math anxiety and TTA scores was found (solid line). As can be seen in Figure 1, the relationship between math anxiety and TTA scores was also linear for the 4 subgroups, being negative for girls in both grades but flat for boys in 2nd grade and positive for boys in 4th grade. It also shows that boys in 4th grade had lower math anxiety scores than the other 3 groups.

### Correlational Analyses

Pearson correlation coefficients were performed between math anxiety scores measured by the CMAQ-R and math test scores (TTA columns 1 to 4 for children in grade 2, and columns 1 to 5 for children in grade 4) separately for girls and boys in each grade. The resulting correlations of the four analyses are presented in Table 3.

**Table 3:** Pearson correlations between math anxiety scores and TTA scores for boys and girls per grade (column 1) and partial Pearson correlations when controlled for test anxiety (column 2).

	**CMAQ-R score**	**CMAQ-R score (partial correlations)**
Boys grade 2: TTA (1 to 4)	−0.04	0.14
Girls grade 2: TTA (1 to 4)	−0.55^**^	−0.37^*^
Boys grade 4: TTA (1 to 5)	0.09	–
Girls grade 4: TTA (1 to 5)	−0.41^*^	–

* *p < 0.05*,

** *p < 0.01*.

As can be seen in Table 3 (column 1), math anxiety scores did not significantly correlate with TTA scores in boys in both grades. These data indicate that in boys math anxiety scores were not related to performance on the TTA. On the other hand, the level of math anxiety significantly correlated with the scores on the TTA in girls in both grades (*p* < 0.01 and *p* < 0.05 for girls in grades 2 and 4 respectively), showing a negative correlation. Girls with higher levels of math anxiety had lower scores on the TTA. Excluding the participants with outliers in the correlation analyses only changed the significance of the girls in grade 4, resulting in a correlation of −0.52 with a *p* < 0.01.

Because test anxiety correlated with math anxiety in children in grade 2 (*r* = 0.56 for girls and *r* = 0.78 for boys, both *p* < 0.001) we performed two partial Pearson correlations with test anxiety as covariate. These analyses showed that the above mentioned correlations did not change substantially for the 2nd grade children while controlling for test anxiety. The correlation between math anxiety and TTA performance was non-significant for boys whereas for girls this correlation remained significant (see column 2, Table 3). Excluding the participants with outliers (one boy for test anxiety and one girl for the TTA) did not change the significances for the partial correlations.

### Moderation Analyses

**Figure 2:**
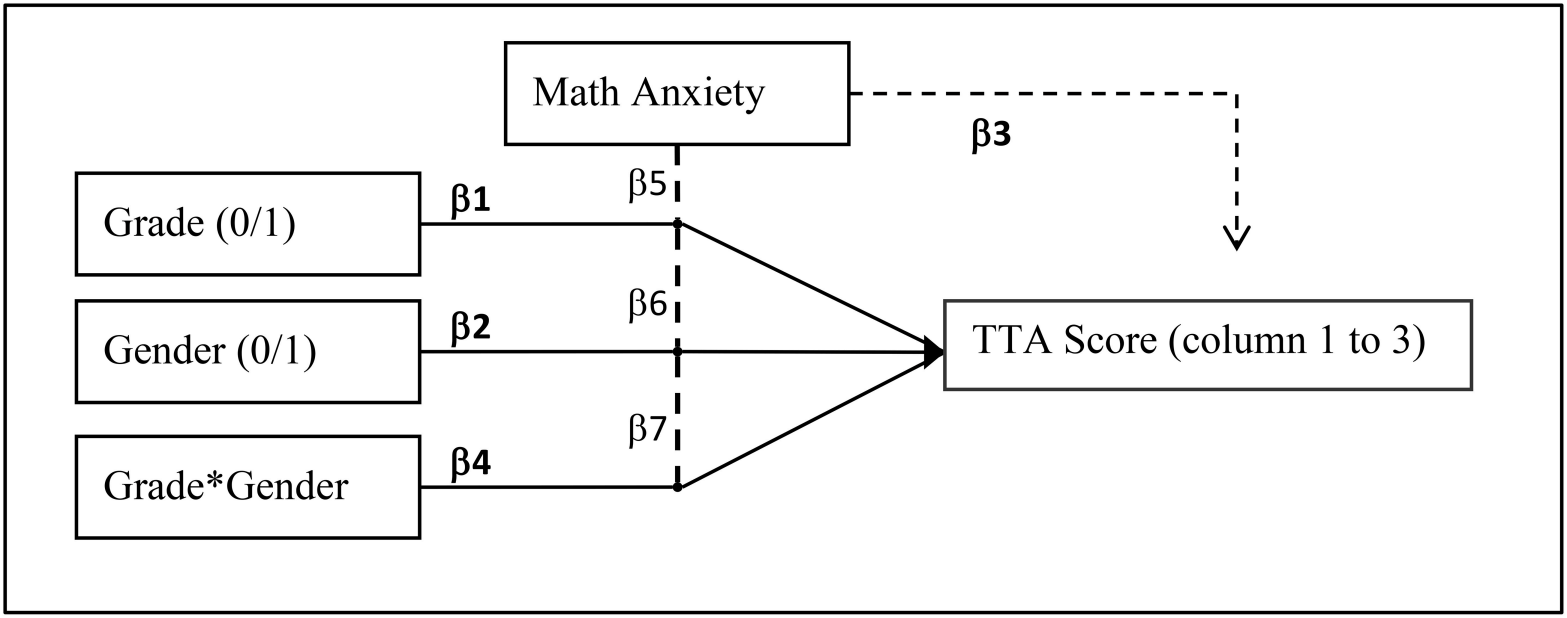
Visual representation of the model. The dotted line indicates the moderation effect of math anxiety, intersecting with grade, gender, and their interaction.

Moderation analyses were conducted to investigate if math anxiety moderated the effects of gender, grade and their interaction on the TTA scores. For the TTA scores we used the scores of columns 1 to 3, because those were performed in the same way in both grades. A moderation model was constructed using the Linear Mixed Procedure in SPSS 24. Data exploration showed the best fit for a model that included all fixed main effects (grade and gender), the interaction between main effects (grade^*^gender), the moderator (math anxiety), and moderation interaction terms (grade^*^math anxiety; gender^*^math anxiety; grade^*^gender^*^math anxiety). Models were compared using Maximum Likelihood (ML) due to the absence of random effects. The final model (see Figure 2), as presented in this paper, includes grade (2 levels: 2nd grade and 4th grade), gender (2 levels: boys and girls), and the mean centered covariate math anxiety (*M* = 31.58, *SD* = 10.01). Math anxiety was mean centered to accurately correct the other effects. The moderation model was checked for Multiple Linearity, Homoscedasticity, Multicollinearity, Outliers, and normality of the residuals. None of the assumptions were violated. The Mixed Procedure was used over the conventional GLM procedure in SPSS because of higher accuracy and convenience. The GLM procedure does not allow for the specification of moderation terms, these would have to be computed manually. The GLM procedure also does not allow for Estimated Marginal Means (EMMs), which provide the *post-hoc* tests. EMMs are available in the Mixed Procedure and allow for comparisons between levels of the predictors on various values of the continuous moderator. A custom UNIANOVA was not used because it does not allow for Maximum Likelihood (ML) estimation.

The moderation model was able to explain 45.1% ($R_{\text{adj}}^{2}$ = 0.451) of the variation in TTA scores, using grade and gender as factors and math anxiety as covariate. Table 4 shows the parameter estimates for the moderation model on the TTA scores (column 1 to 3). The parameter estimates indicated main effects for grade (*B* = −14.988, *p* < 0.001), gender (*B* = 9.203, *p* = 0.007), and math anxiety (*B* = −0.498, *p* = 0.037). Children in 2nd grade had significantly lower TTA scores than children in 4th grade (boys *M*_2nd−4thgrade_ = −24.81, *p* < 0.001; girls *M*_2nd−4thgrade_ = −14.99, *p* < 0.001). As stated before this was expected because we used raw scores. With regard to the significant grade by gender interaction (*B* = −9.819, *p* = 0.034) estimated marginal means were ordered by using the coefficient and formula provided by the model. It was found that the difference between boys and girls was not significant for 2nd graders (2nd grade; *M*_boys−girls_ = −0.616, *p* = 0.845), but was significant for 4th graders (4th grade; *M*_boys−girls_ = 9.204, *p* = 0.007). Math anxiety did not moderate the effect of grade (*B* = −0.041, *p* = 0.891), nor did it moderate the grade by gender interaction (*B* = −0.336, *p* = 0.492). However a significant moderation effect of math anxiety on gender was found (*B* = 0.875, *p* = 0.026).

**Table 4:** Parameter estimates for the moderation model on the TTA scores.

	**Unstandardized coefficients**		**Standardized coefficients**			**95% CI**	
	**B**	**Std.Error**	**Beta**	***t***	**Sig**	**LB**	**UB**
(Constant)	58.180	2.144		27.136	0.000	53.937	62.424
Grade	−14.988	3.004	−0.910	−4.989	0.000	−20.935	−9.041
Gender	9.203	3.332	0.539	2.762	0.007	2.608	15.799
Math anxiety	−0.498	0.237	−0.303	−2.104	0.037	−0.966	−0.029
Grade* Gender	−9.819	4.581	−0.596	−2.144	0.034	−18.886	−0.752
Grade*MA	−0.041	0.295	−0.025	−0.138	0.891	−0.625	0.544
Gender*MA	0.875	0.388	0.532	2.255	0.026	0.107	1.642
Grade*Gender*MA	−0.336	0.487	−0.204	−0.689	0.492	−1.301	0.629

*LB, lower bound; UB, upper bound; MA, Math Anxiety*.

#### Gender by Math Anxiety Interaction

The gender by math anxiety interaction was further explored using estimated marginal means, computing the mean TTA score per gender with low math anxiety (1 SD below the mean), average MA, and high math anxiety (1 SD above the mean) (see Table 5). As for the numerosity of the math anxiety groups, this was equal to the total N of the dataset as no groups were created. Instead of dividing the dataset into low, average, and high math anxiety groups, we opted for a regression method. The analysis estimated the scores of low, average, and high groups using the regression model based on all individuals. These three estimates were based on the standard deviation of the math anxiety scale. Scores for low math anxiety were estimated using the value of math anxiety equal to 1 SD below the mean, for average math anxiety the value of the mean was used, and for high math anxiety the value equal to 1 SD above the mean. This was done to increase the power of the analysis and to avoid having to split up the data into unequal groups based on an arbitrary cut-off value.

**Table 5:** Estimated marginal means for the gender by math anxiety interaction for the TTA scores.

	**95% confidence interval**					
**Math anxiety**	**Gender**	**Mean**	**Std. Error**	**df**	**Lower bound**	**Upper bound**
Low (−1 SD)	Boys	53.091_a_	2.219	124	48.700	57.483
	Girls	55.875_a_	2.175	124	51.571	60.179
Average	Boys	54.980_b_	1.729	124	51.558	58.402
	Girls	50.686_b_	1.502	124	47.713	53.659
High (+1 SD)	Boys	56.869_c_	2.932	124	51.065	62.672
	Girls	45.497_c_	2.038	124	41.463	49.531

a Mean Estimated with MA = −10.01;

b Mean Estimated with MA = 0;

c *Mean Estimated with MA = 10.01*.

**Figure 3:**
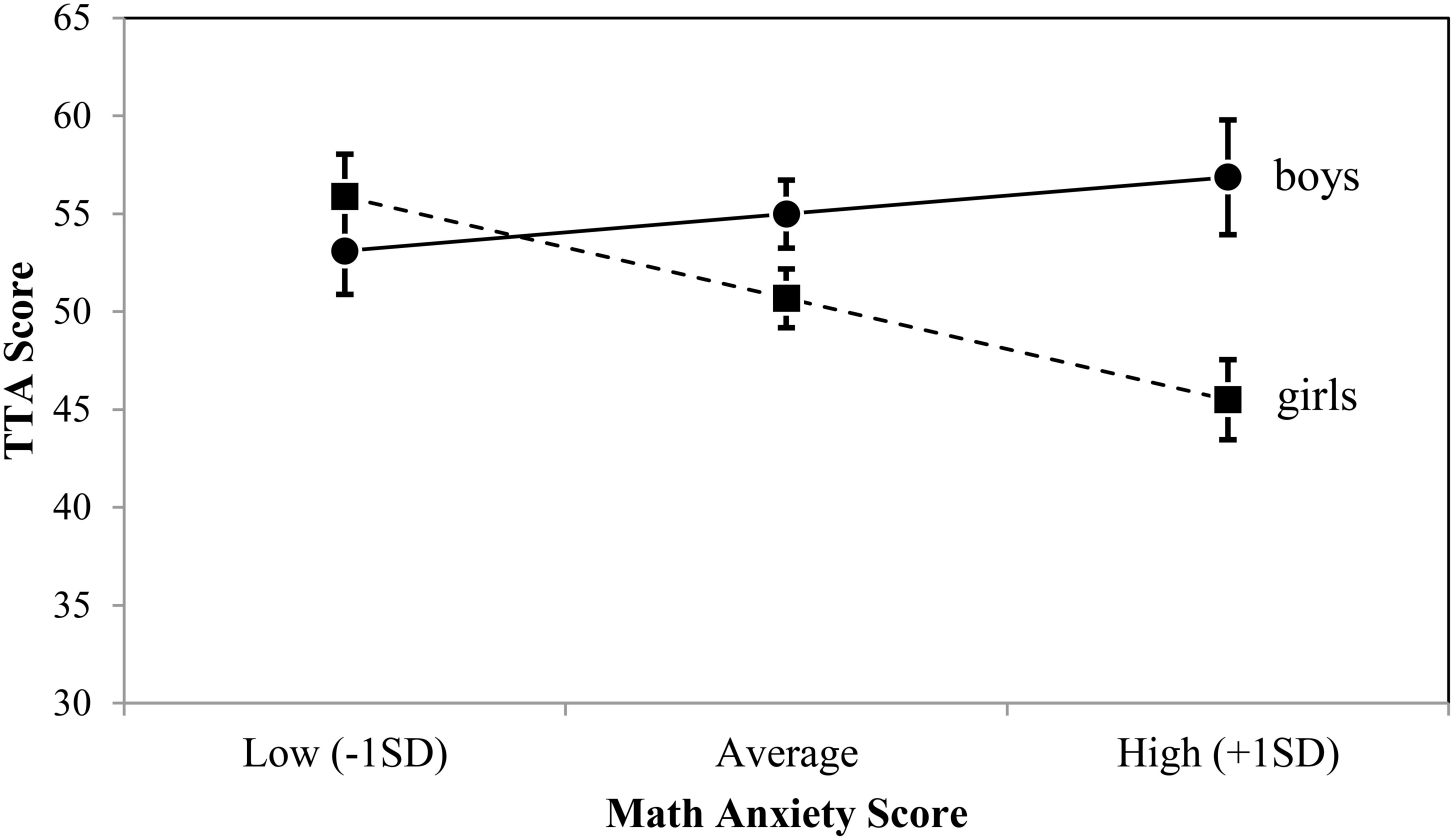
The gender by math anxiety interaction, with TTA scores estimated per gender for low, average, and high math anxiety scores.

The difference in TTA scores between boys and girls is displayed in Figure 3. Math anxiety had a significant negative slope for girls (*B* = −0.498, *p* = 0.027), while boys did not show a significant effect of math anxiety (*B* = 0.377, *p* = 0.260). When math anxiety scores were low (1 SD below the mean) the difference in TTA scores between boys and girls was not significant being only 2 points (*M*_Boys−Girls_ = −2.784, *p* = 0.372). At an average math anxiety level the difference in TTA scores between boys and girls was larger, about 4 points, and showed a trend (*M*_Boys−Girls_ = 4.294, *p* = 0.063). When the math anxiety score was 1 SD above the mean, the difference in TTA scores between boys and girls was about 11 points, showing a significant difference between the genders (*M*_Boys−Girls_ = 11.372, *p* = 0.002).

#### Gender by Grade by Math Anxiety Interaction

**Figure 4:**
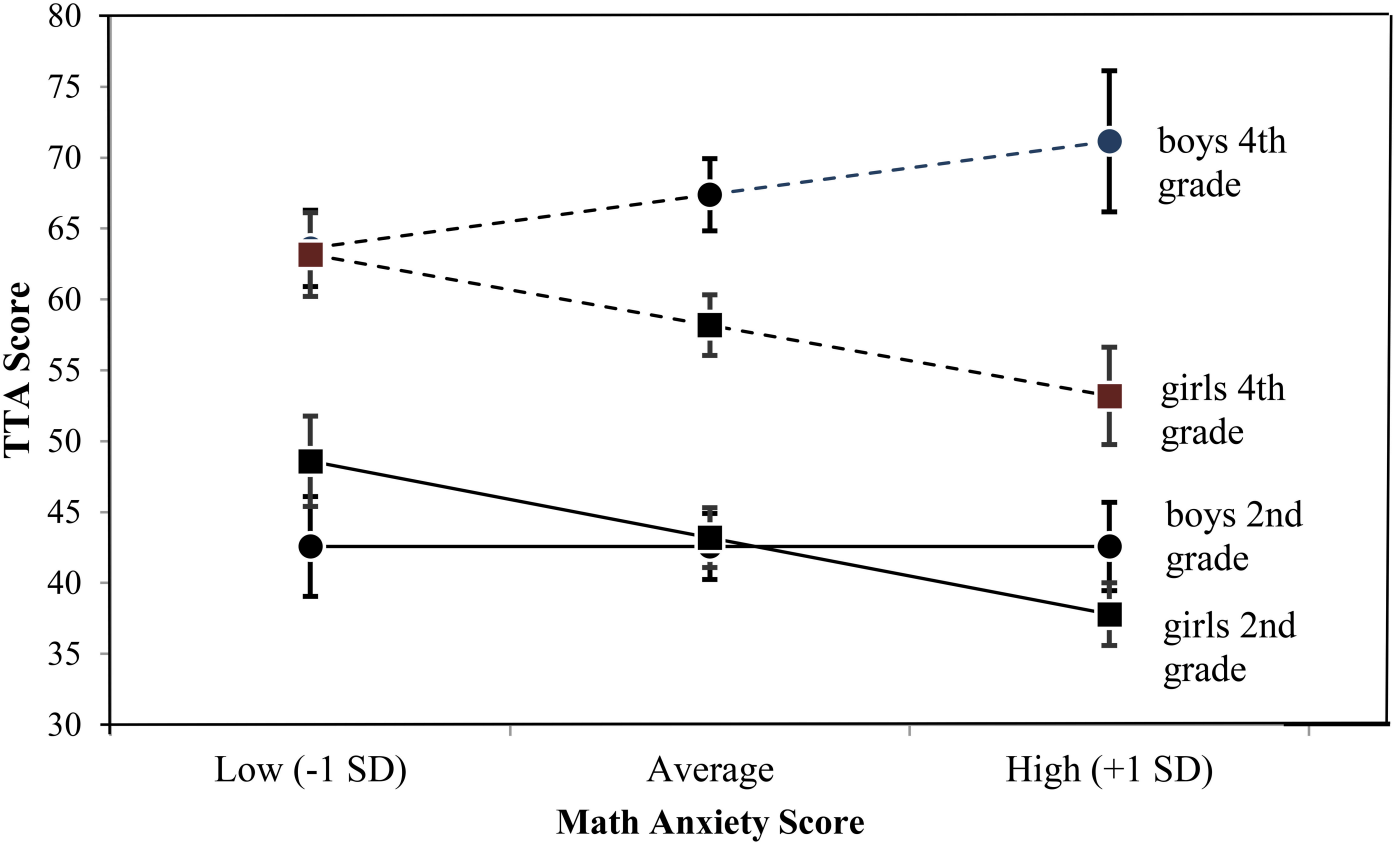
The gender by grade by math anxiety interaction, with TTA scores estimated per gender and grade for low, average, and high math anxiety scores.

Although the three-way interaction was not significant, because we observed different significance levels in the correlations for girls in 2nd and 4th grades, we split the data on gender and grade. The estimated marginal means are shown in Table 6. When split on gender and grade it can be seen in Figure 4 that math anxiety had a negative slope for girls in 2nd grade which was significant (*B* = −0.539, *p* = 0.001), while boys in 2nd grade did not show any effect of math anxiety (*B* = 0, *p* = 0.998). This also accounted for the significant gender by math anxiety interaction that was found in the 2nd graders when the data were split on grade only. The significant negative effect of math anxiety for girls was less pronounced for girls in 4th grade, although showing a trend toward significance (*B* = −0.498, *p* = 0.053). A non-significant effect of math anxiety was observed for boys in 4th grade (*B* = 0.377, *p* = 0.353). Looking at the difference between gender, per grade and MA, it can be seen in Figure 4 that only in 4th graders a significant gender effect occurred at an average (*p* = 0.007) and high (*p* = 0.003) math anxiety score, while this gender effect was not observed in the 2nd graders. Due to the nature of the slopes and the results from the overall tests it seems that this is most likely a false positive, or at least an inflation of the effects. As can be seen in Figure 1, most boys in 4th grade scored low or average for MA. With the slopes being non-significant for boys in both grades, this means they are most likely 0 in the population.

**Table 6:** Estimated marginal means for the gender by grade by math anxiety interaction for the TTA scores.

	**95% confidence interval**					
**Math anxiety**	**Gender**	**Grade**	**Mean**	**Std. Error**	**Lower bound**	**Upper bound**
Low (−1 SD)	Boys	2nd grade	42.573_a_	3.527	35.592	49.554
		4th grade	63.610_a_	2.693	58.280	68.940
	Girls	2nd grade	48.585_a_	3.191	42.268	54.902
		4th grade	63.166_a_	2.955	57.318	69.013
Average	Boys	2nd grade	42.576_b_	2.334	37.956	47.197
		4th grade	67.384_b_	2.551	62.334	72.433
	Girls	2nd grade	43.192_b_	2.105	39.026	47.358
		4th grade	58.180_b_	2.144	53.937	62.424
High (+1 SD)	Boys	2nd grade	42.580_c_	3.113	36.419	48.741
		4th grade	71.157_c_	4.970	61.321	80.994
	Girls	2nd grade	37.799_c_	2.217	33.410	42.188
		4th grade	53.195_c_	3.420	46.426	59.964

a Mean Estimated with MA = −10.01;

b Mean Estimated with MA = 0;

c *Mean Estimated with MA = 10.01*.

## Discussion

This study addressed the question if math anxiety differentially affected and moderated math performance in boys and girls in grades two and four. To our knowledge this has not been studied before in early elementary school children. We indeed found gender related differences. Despite the fact that boys and girls had more or less similar levels of math anxiety and comparable scores on the math test we found differences between boys and girls regarding the correlation between math anxiety and math performance. In both grades, this correlation was significant and negative for girls but not for boys. A higher level of math anxiety was a negative predictor of math performance for girls only. Analysis addressing the question if math anxiety moderated the effect of gender and grade and the interaction of gender by grade on math performance showed that math anxiety significantly moderated the effect of gender on the TTA scores but not the interaction of gender and grade. Math anxiety moderated the effect of gender on the TTA, but the difference in TTA scores between both genders was only significant when math anxiety scores were high. Furthermore, only in girls the level of math anxiety moderated the TTA score significantly and negatively, with lower TTA scores observed in girls with higher math anxiety levels. Math anxiety levels had no influence on TTA scores in boys. The current results show that math anxiety is already negatively linked to math performance in girls as early as 2nd grade.

Regarding math performance, we found that girls and boys performed equally on the math test, although boys performed slightly better at the TTA when only results of columns 1 to 3 were taken into account or all columns for children in grade four. This finding was replicated in the moderation analysis. Small or lacking gender differences in math performance is in line with recent research showing that gender differences with respect to math performance in children are decreasing or non-existent. Hyde et al. (2008) looked at gender differences in math performance in over 7 million students from grades two through eleven based on state assessments of 10 USA states. They report an absence of gender differences, even for hard items requiring substantial depth of knowledge. Similar math performance for boys and girls in elementary grades has been described by others (Ho et al., 2000; Beilock et al., 2010; Dowker et al., 2012; Hill et al., 2016), also with respect to scores on the TTA (Erturan and Jansen, 2015; Schleepen and Van Mier, 2016). Lindberg et al. (2010) found gender differences in math performance to be negligible for elementary-school and middle-school aged children in a meta-analysis including studies published between 1990 and 2007. However, if gender differences are reported they are usually observed during high school years (Hyde et al., 1990; Liu et al., 2008; Rosselli et al., 2009; Lindberg et al., 2010).

We found significant main effects of grade for scores on the TTA and levels of MA. Children in grade 4 solved more problems in the TTA (columns 1 to 3) than children in grade 2. The significant grade-related performance differences in the TTA are in line with findings by Jansen and colleagues in 3rd to 6th graders (Jansen et al., 2013) and in 3rd to 8th graders in the study by Erturan and Jansen (2015). Because all children performed the same arithmetic task and raw scores were used, this result was as expected. However, we found a significant effect of grade regarding the level of math anxiety. Children in 2nd grade reported to be more math anxious than children in 4th grade. These findings are in contrast with results by others who reported similar math anxiety scores for 1st and 2nd grade children (Ramirez et al., 2013), 2^nd^ and 3^rd^ graders (Young et al., 2012) and for children in grade 3 through 6 (Erturan and Jansen, 2015). The mean score on the CMAQ-R for the second graders (34.2) is however comparable to the mean scores of 38.4 and 38.6 on this test for first and second graders in the study of Maloney et al. (2015) and Ramirez et al. (2016), respectively. Because the CMAQ-R is adapted for use in first grade, it is possible that in particular the math related problems in this test were much easier for the 4th graders than the 2nd graders and therefor elicited less math anxiety in the former group.

We did not find a significant gender difference in math anxiety in our study, consistent with findings of others in elementary school children (Dowker et al., 2012; Young et al., 2012; Jansen et al., 2013; Ramirez et al., 2013; Erturan and Jansen, 2015; Schleepen and Van Mier, 2016; Kucian et al., 2018). This is contradictory to results by Hembree (1990) who reported gender differences regarding math anxiety in his meta-analysis from grade 6 to college. Similar gender differences were also observed in junior and senior high school students (Devine et al., 2012; Goetz et al., 2013; Bieg et al., 2015). These contrasting findings might be due to the fact that math anxiety increases in high school (Hembree, 1990) with a likely increase in the gender gap as well. However, Ma and Xu (2004) found comparable levels of math anxiety for male and female students in junior and senior high school. Contrasting results might also be due to the use of variations in sample characteristics, different math anxiety tests or scales or the type of math anxiety assessed (Goetz et al., 2013; Erturan and Jansen, 2015; Jansen et al., 2016). Additionally, we found a trend toward significance regarding differences in math anxiety between girls and boys in the current study, with girls reporting slightly higher levels of math anxiety. When the outlier, the boy with a high math anxiety score, was excluded from the analyses, we found a significant gender effect of math anxiety, with girls reporting significantly more math anxiety.

As for the relation between math anxiety and math performance, the moderation analyses showed that math anxiety had a significant and negative slope regarding TTA scores for girls, with non-significant slopes for boys. For girls the slope was highly significant for 2^nd^ graders and almost significant for 4th graders. To establish if this is a developmental effect, in future studies children in different and higher grades should be included. The results of the correlation analyses also showed that math anxiety was not related to math performance in boys. Although our data might suggest that having more math anxiety results in better math performance in boys in 4th grade, the positive slope and positive correlation were however not significant. As we already mentioned in the results section, this is most likely a false positive, due to the fact that in our sample most boys in 4th grade had lower MA scores compared to the other groups. As for girls, math anxiety was negatively correlated to performance on the overall score on the TTA. This correlation was still significant in 2nd grade girls when the scores of the test anxiety questionnaire were added as a covariate in the correlational analyses. A limitation of the current study is the fact that we could not measure test anxiety in the children in grade 4, and therefore do not know if this would also have been the case for girls in grade 4. However, a significant correlation between math anxiety and performance on the TTA after controlling for test anxiety in girls in grade 5 was reported by Schleepen and Van Mier (2016). It is therefore reasonable to assume that this might also have been the case for girls in grade 4 in the current sample. These results show that even after controlling for test anxiety, math anxiety negatively affected math performance in girls.

Our finding that math anxiety negatively correlated with math performance only in girls is in line with other studies regarding elementary school children (Erturan and Jansen, 2015; Schleepen and Van Mier, 2016) and extends previous research by showing that an inverse relation between math anxiety and math performance can already been found in girls as early as second grade. Studies including early elementary school children have reported a negative relationship between math anxiety and math performance (Ramirez et al., 2013, 2016), however these studies did not take gender into account or did not report data on gender. In line with their results, we also found a negative slope between TTA scores and math anxiety in our study when we did not take gender into account (see Figure 1, solid line). Wu et al. (2012) found that math anxiety scores in younger children (2nd and 3rd graders) correlated significantly with mathematical reasoning scores, involving more complex verbal mathematical problem solving. Math anxiety scores, however, did not significantly correlate with scores on numerical operations requiring basic symbolic processing and fact retrieval like simple equations involving basic operations of addition, subtraction, multiplication and division. As the authors state, the children in their study were given unlimited time to solve the numerical operations problems and did not have any time pressure. Although the task in the current study also involved basic operations of addition, subtraction, multiplication and division, it was performed under time pressure. Caviola et al. (2017a) reviewed studies addressing the effects of time pressure on math performance and concluded that time pressure indeed affected math performance by choosing strategies that can be applied quickly but might be suboptimal. Gender related effects were not studied or reported. The authors found only one study involving elementary school children that included a time pressure manipulation to test the effect of math anxiety on math performance in boys and girls. The study by Tsui and Mazzocco (2006), involving 6th graders, showed a significant difference in math performance between timed and untimed testing only for girls but not for boys regardless of the level of MA. Girls were less accurate during timed than during untimed testing. In line with this finding, it is possible that time pressure mainly affected math performance in girls in the current study. The time pressure might have elicited even more math anxiety in math anxious girls resulting in lower performance on the TTA. Future studies should also include a condition without time pressure and/or measure math anxiety before, during and after the TTA to check if these factors are linked to the gender related differences.

Results of the current study suggest that having to solve problems under time pressure affected mainly girls who are more math-anxious, which especially affected performance in the youngest girls. Researchers have reported that math anxiety often leads to avoidance of math related activities and classes resulting in less experience with and exercise of math leading to lower math performance (Hembree, 1990; Ashcraft, 2002; Dowker et al., 2016). Jansen et al. (2016) found that even in highly educated women math anxiety was not only negatively related to math skills but also to the use of math in everyday life. This stresses the importance of studying and preventing math anxiety in younger girls.

Of consideration is that we did not include a measure of working memory. Studies involving adults have shown that in particular participants with high working memory are more affected when performing under pressure most likely resulting in math anxiety (Beilock and Carr, 2005; Sattizahn et al., 2016). Studies examining this relation in children found a negative relation between math anxiety and math performance only for children who were higher in working memory (Ramirez et al., 2013; Vukovic et al., 2013) and showed that high math anxious children with high working memory capacity avoided the use of advanced problem solving strategies resulting in lower math performance than children with high working memory who were low in math anxiety (Ramirez et al., 2016). Future studies should include a measure of working memory to establish if the gender related findings in the current study are due to differences in working memory capacity.

It is unclear why particularly math performance in girls is more affected by math anxiety. As Schleepen and Van Mier (2016) have speculated, it is possible that math anxiety evokes worrisome thoughts and that these ruminations take up available working memory capacity reducing the capacity that is necessary to solve the arithmetic problems. According to the attentional control theory (Eysenck et al., 2007) ongoing working memory processes needed to solve (math) problems are disrupted in (math) anxious individuals because they allocate attention to the intrusive thoughts and worries, instead of to the (math) problems (Ashcraft and Faust, 1994; Ashcraft and Kirk, 2001). Either math-anxious girls might have more ruminations than math-anxious boys or the latter are better able to inhibit paying attention to these unwanted contemplations than the former (Hopko et al., 1998).

Exposure to negative attitudes about math by role models, like parents and teachers, has been established as a possible source of lower math performance and development of math anxiety in children. Recently, a relation between parents' math anxiety and math performance of their children has been established (Casad et al., 2015; Maloney et al., 2015). Casad et al. (2015) showed that parents' math anxiety was related to their children's math anxiety and that both interacted with math education outcomes of the children. They additionally looked at the interaction of children's math anxiety with parents' math anxiety within gendered dyads and found that this relationship was most significant in same-gender parent-child dyads, whereas the mother-daughter dyad was most prominent. They found that when both mother and daughter had high math anxiety, daughters had more negative math outcomes. Maloney et al. (2015) found that when math anxious parents frequently helped their children with math problems, math performance of their children decreased during the school year while math anxiety increased. Next to parents, also math anxious school teachers have been shown to negatively affect math attitudes in their students (Beilock et al., 2010). The authors found that math-anxious female 1st and 2nd grade teachers, who had negative attitudes about math, passed these feelings on to girls but not to boys. Also teachers' attitudes toward math and their behavior toward their students might invoke math anxiety in children, for instance by showing a gender bias, or embarrassing students in front of peers in class (Jackson and Leffingwell, 1999; Ashcraft, 2002; Furner and Duffy, 2002). When teachers belief that girls lack understanding and competence in math this might affect their help and support, which in turn might lead to less engagement and perceived ability regarding math in girls (Park et al., 2014). Additionally, it was found that certain instructional methods in math education were related to math anxiety (Brady and Bowd, 2005).

Another option for the gender-related difference between math anxiety and math performance might be that math-anxious girls perform less well as a consequence of exposure to gender stereotypes, the so-called stereotype threat. With regard to math this entails the stereotype that males are better at math than females. Gender stereotypes about math have been found to be evident as early as first grade (Cvencek et al., 2011). When girls and females fear that their math performance might confirm this negative stereotype this can lead to math anxiety (Maloney et al., 2014; Dowker et al., 2016). The idea is that this threat evokes negative thoughts and ruminations, leaving less working memory resources available for the math task at hand.

Studying the effect of gender in relation to math anxiety in younger children will not only increase the understanding of the relation between math anxiety and math performance in both genders but is a critical step in developing interventions to decrease math anxiety in children and in particular in girls. It is vital that parents and teachers are aware of the detrimental effect of math anxiety on math performance in girls. Teachers and parents should be aware of the stereotype threat, should prevent gender stereotyping (Casad et al., 2015) and develop strategies to counteract or nullify the stereotype threat (Schmader et al., 2004; Thoman et al., 2008). Additionally, instead of modeling negative attitudes about math to children, they should model positive attitudes (Beilock and Maloney, 2015; Dowker et al., 2016). Interventions to reappraise upcoming math tasks and perceive them as a challenge instead of a threat have been shown to be effective (Jamieson et al., 2010; Mattarella-Micke et al., 2011). Others have suggested that education interventions that emphasize the control of negative emotional responses to math stimuli may decrease math anxiety and increase math performance (Lyons and Beilock, 2012). Interventions by adapting arithmetic problem difficulty to children's ability levels showed an improvement in math performance which was most pronounced in conditions with the highest success rate and were higher for girls than for boys (Jansen et al., 2013). It goes beyond saying that strategies should be applied and interventions should be started as early as possible to prevent that children, and especially girls, develop math anxiety and in turn avoid math related tasks and courses and as a consequence might decrease their options regarding career choices and paths later in life.

## Conclusion

We have shown that despite similar math performance and math anxiety scores in boys and girls, math anxiety significantly and negatively affected math performance only in girls. In the youngest girls this effect was still significant after controlling for test anxiety for the TTA, an arithmetic test that had to be performed under time pressure. Our results highlight the importance of taking gender differences into account when studying the effect of math anxiety. Considering the lifelong consequences of math anxiety on math performance and subsequent avoidance of STEM careers it is important to prevent the development of math anxiety in young children, especially in girls. For girls who have encountered the negative consequences of math anxiety on math performance it is imperative to reduce the level of math anxiety. Therefore more emphasis should be directed at studying gender differences related to math anxiety. Additionally, intervention studies to reduce math anxiety should take into account and stress gender related effects of the interventions.

## Author Contributions

HV developed the study design, performed the data analyses, and drafted the manuscript. TS developed the study design, collected the data, and helped with editing the manuscript. FV helped with data analyses and with editing the manuscript.

### Conflict of Interest Statement

The authors declare that the research was conducted in the absence of any commercial or financial relationships that could be construed as a potential conflict of interest.
